# Resting State fMRI Functional Connectivity Analysis Using Dynamic Time Warping

**DOI:** 10.3389/fnins.2017.00075

**Published:** 2017-02-17

**Authors:** Regina J. Meszlényi, Petra Hermann, Krisztian Buza, Viktor Gál, Zoltán Vidnyánszky

**Affiliations:** ^1^Department of Cognitive Science, Budapest University of Technology and EconomicsBudapest, Hungary; ^2^Brain Imaging Centre, Research Centre for Natural Sciences, Hungarian Academy of SciencesBudapest, Hungary

**Keywords:** functional magnetic resonance imaging, classification, Dynamic Time Warping, resting state connectivity, connectome

## Abstract

Traditional resting-state network concept is based on calculating linear dependence of spontaneous low frequency fluctuations of the BOLD signals of different brain areas, which assumes temporally stable zero-lag synchrony across regions. However, growing amount of experimental findings suggest that functional connectivity exhibits dynamic changes and a complex time-lag structure, which cannot be captured by the static zero-lag correlation analysis. Here we propose a new approach applying Dynamic Time Warping (DTW) distance to evaluate functional connectivity strength that accounts for non-stationarity and phase-lags between the observed signals. Using simulated fMRI data we found that DTW captures dynamic interactions and it is less sensitive to linearly combined global noise in the data as compared to traditional correlation analysis. We tested our method using resting-state fMRI data from repeated measurements of an individual subject and showed that DTW analysis results in more stable connectivity patterns by reducing the within-subject variability and increasing robustness for preprocessing strategies. Classification results on a public dataset revealed a superior sensitivity of the DTW analysis to group differences by showing that DTW based classifiers outperform the zero-lag correlation and maximal lag cross-correlation based classifiers significantly. Our findings suggest that analysing resting-state functional connectivity using DTW provides an efficient new way for characterizing functional networks.

## Introduction

Resting-state fMRI (Biswal et al., 1995, 2010) has become increasingly popular in the last decade. The technique requires no special MR compatible hardware or software equipment and yet is an effective tool for the exploration of various functional networks in the brain (Biswal et al., 1995; Fox et al., 2005; Fox and Raichle, 2007; Margulies et al., 2010; Thomas Yeo et al., 2011; Kalcher et al., 2012). The synchronized spontaneous low frequency fluctuations of the BOLD signal within a task-free environment are known to represent the functional connections of different brain areas (Fox and Raichle, 2007).

Traditionally, the analysis of resting-state networks across a single scanning session employs techniques that assume temporal stationarity and zero-lag synchrony, so that measures of linear dependence can be computed over the entire scan and used to characterize the strength of connections across regions. The most popular approaches for the evaluation of functional connectivity are the anatomically or functionally confined seed-based correlation analysis (SCA) and the less restrictive independent component analysis (ICA), but several other valuable methods have been developed as well (Smith et al., 2011). For SCA (Biswal et al., 1995), a whole-brain connectivity map is obtained by iteratively calculating the linear correlation between the time series of a seed region or voxel and every other region or voxel specified using correlation coefficient as the measure of similarity. ICA (McKeown et al., 1998) is a data-driven technique, which identifies a predefined number of spatial templates and corresponding time courses to model the data: the activities of the individual voxel are linear combination of these component time series. They also determine the main source or sources that affect the BOLD time course of the corresponding voxel, thus resulting in a fuzzy clustering of voxels. Each independent source defines a component, therefore clusters arise not only from actual neural synchronization, but from other signal sources, like measurement noise and artifacts.

However, evidence from a growing number of fMRI (Chang and Glover, 2010; Sakoğlu et al., 2010; Kiviniemi et al., 2011; Handwerker et al., 2012; Jones et al., 2012; Smith, 2012; Allen et al., 2014), near-infrared spectroscopic and MEG studies (de Pasquale et al., 2010; Brookes et al., 2011; Keilholz, 2014; Li et al., 2015) suggests that functional connectivity exhibits dynamic changes within time scales of seconds to minutes. These studies have moved beyond the conventional static, zero-lag correlation analysis either to capture static lag effects (e.g., with maximal lag cross-correlation: Jafri et al., 2008) or time-varying changes in functional connectivity (Allen et al., 2014). It is claimed that dynamic functional connectivity measures may indicate changes in macroscopic neural activity patterns underlying critical aspects of the human cognitive functions. However, limitations with regard to analysis and interpretation remain (Hutchison et al., 2013; Keilholz, 2014). Sliding-window analysis (Kiviniemi et al., 2011; Handwerker et al., 2012; Hutchison et al., 2013) and other approaches such as time–frequency coherence analysis or spontaneous co-activation patterns analysis (CAP; Liu and Duyn, 2013; Chen et al., 2015) have been suggested to capture dynamics and temporal-lag structures. The latter applies clustering analysis to generate resting state networks from the data.

Furthermore, time-frequency analysis based on wavelet transform coherence (WTC) indicated that coherence and phase difference (delay, temporal lag) between and within identified resting-state networks is indeed variable in time (Chang and Glover, 2010). This study showed that classic anticorrelation patterns between default-mode network (DMN) and regions with negative correlations (“anticorrelated,” “task-positive” TPN regions) appeared to be transient rather than stable phenomenon. Intermittent increase in coherence could be observed with 180° phase difference (within 0.01–0.05 Hz range equivalent to ~10–50 s phase delay). This could explain that the magnitude of negative correlations between the two networks have been reported to be weaker and much less consistent in comparison with the positive correlations measured between nodes within the same network (Shehzad et al., 2009). The relative weakness of negative correlations may also be explained with the presence of common (physiologic) noise across the brain due to e.g., respiration and cardiac processes (Wise et al., 2004; Birn et al., 2006; Shmueli et al., 2007; Chang et al., 2009). This noise can bias inter-regional correlation coefficients in the positive direction; and both SCA and ICA are inherently sensitive to an additive noise term. Complex preprocessing strategies were developed to suppress the effect of non-neural components (Shirer et al., 2013). Global signal regression (GSReg) can effectively reduce artifacts arising from common sources for all brain voxels (Desjardins et al., 2001); however, it may also introduce artificial anticorrelations, which do not reflect any real neural activity (Chang and Glover, 2009; Fox et al., 2009; Murphy et al., 2009; Saad et al., 2012). ICA based analysis is also affected: for practical reasons, ICA is always preceded by principal component analysis (PCA) to reduce dimensionality, and PCA subtracts the global signal from the time series of every voxel.

Both within- and between-subject reliability of the resulting connectivity strengths is heavily studied (Zuo et al., 2010; Guo et al., 2012; Shirer et al., 2013; Zuo and Xing, 2014). Moreover, the consensus on what particular preprocessing steps and parameter sets are to be used is also lacking. Numerous groups apply GSReg in their preprocessing thread explicitly (Anticevic et al., 2014; Cousijn et al., 2014; Dodhia et al., 2014) or implicitly with the application of ICA decomposition. Furthermore, another important yet often neglected assumption of functional connectivity analysis is that the measurements are independent, therefore the order of these measurements should not matter. This assumption is obviously violated in case of fMRI, therefore autocorrelations within the analyzed time series distort the results. Although distortions (inflation of linear correlation significance) can be reduced via prewhitening techniques following nuisance regression, only a limited number of studies apply prewhitening to resting state fMRI data (Christova et al., 2011; Arbabshirani et al., 2014; Bright et al., in press). Standard ICA is similarly unable to correctly handle autoregressive and autoregressive moving average processes, where the measured value of a time series at a given point linearly depends on previous values, however some approaches already exist to deal with this problem (Lee et al., 2011).

To address the abovementioned issues, we propose an alternative measure of similarity between BOLD signals called Dynamic Time Warping (DTW) distance (Sakoe and Chiba, 1978). DTW performs a non-linear warping on the compared time series, therefore it can correct for non-stationary time-lags introduced by the dynamic switching of brain states (Allen et al., 2014; Chen et al., 2015) and shape distortions between brain regions that can arise from the variability of the shape of the haemodynamic response function. Consequently, DTW is able to account for intermittent phase delays ranging from 0 to 5 s reported in positive correlations to 30–100 s lags interpreted as anticorrelations in low frequency fluctuations. More importantly, DTW has been developed specifically for time series analysis and classification (Sakoe and Chiba, 1978; Xi et al., 2006), therefore it is able to handle autocorrelations induced by colored noise components. Unlike the conventional approaches for fMRI data analysis, DTW provides a single (scalar) measure for non-stationary process pairs with unstable non-zero temporal lag structure, thus characterizing a wide range of connections and still enabling simple multi-subject statistics. Furthermore, DTW distance is also less sensitive to linearly combined common noise in the data. Based on these properties, we hypothesize that DTW based connectivity analysis yields results more stable than conventional approaches, while the fact that DTW distance can capture complex relationships implies its advantage as feature in connectivity based classification experiments (Meszlényi et al., 2016a,b). Although DTW has been used to analyze EEG data (Huang and Jansen, 1985; Gupta et al., 1996; Karamzadeh et al., 2013), its application to fMRI data was limited to preprocessing (Pickering and Garrod, 2014; Silbert et al., 2014; Dinov et al., 2016), e.g., Dinov et al. applied DTW in a novel spectrum calculation framework and Silbert et al. used DTW to warp all BOLD signals with the same warping path according to a speech signal, and the warped fMRI data was further analyzed with correlation. In contrast, our study applies DTW distance directly to measure similarity between BOLD signals, and warps each pair of time-series to each other.

In the present study, we investigated the feasibility of DTW for resting-state functional connectivity as measured with fMRI. To validate our approach, we created three experiments: in Experiment 1 we demonstrate the strengths and possibilities of DTW based functional connectivity calculation with two simulations, Experiment 1.A examines the effect of transient interactions on DTW distance and correlation coefficient values, while Experiment 1.B reveals how DTW distance and correlation coefficients behave in the presence of common noise (e.g., global signal). Experiment 2 proves that DTW is applicable to real fMRI data. For that purpose we measured 20 runs of resting state fMRI on a single subject to study the reliability of connectivity patterns within subject. Experiment 2.A reveals how global signal regression effects seed based connectivity maps based on DTW, zero-lag correlation or maximal-lag cross-correlation calculation, while in Experiment 2.B we examine the three metrics robustness for multiple measurements by comparing the results from the 20 resting state runs that were measured within a week. In Experiment 3, we quantitatively compare the three metrics' (DTW, zero-lag correlation and maximal-lag cross-correlation) sensitivity for group differences in a classification pipeline on a public dataset containing five runs of resting state fMRI on 26 subjects.

With these experiments we demonstrate that connectivity maps resulted from hypothesis-driven (seed-based) DTW connectivity analysis are consistent with previous findings using conventional approaches. We also show that DTW-based connectivity maps are more robust across preprocessing pipelines as well as across repetitions, than those calculated by means of SCA or maximal lag cross-correlation. Furthermore, our findings demonstrate that DTW reveals even subtle group differences that SCA cannot extract: the same (LASSO type) classifier achieved significantly higher accuracy when it relies on DTW distance features compared to correlation or cross-correlation values.

## Materials and methods

### The dynamic time warping algorithm

**Figure 1:**
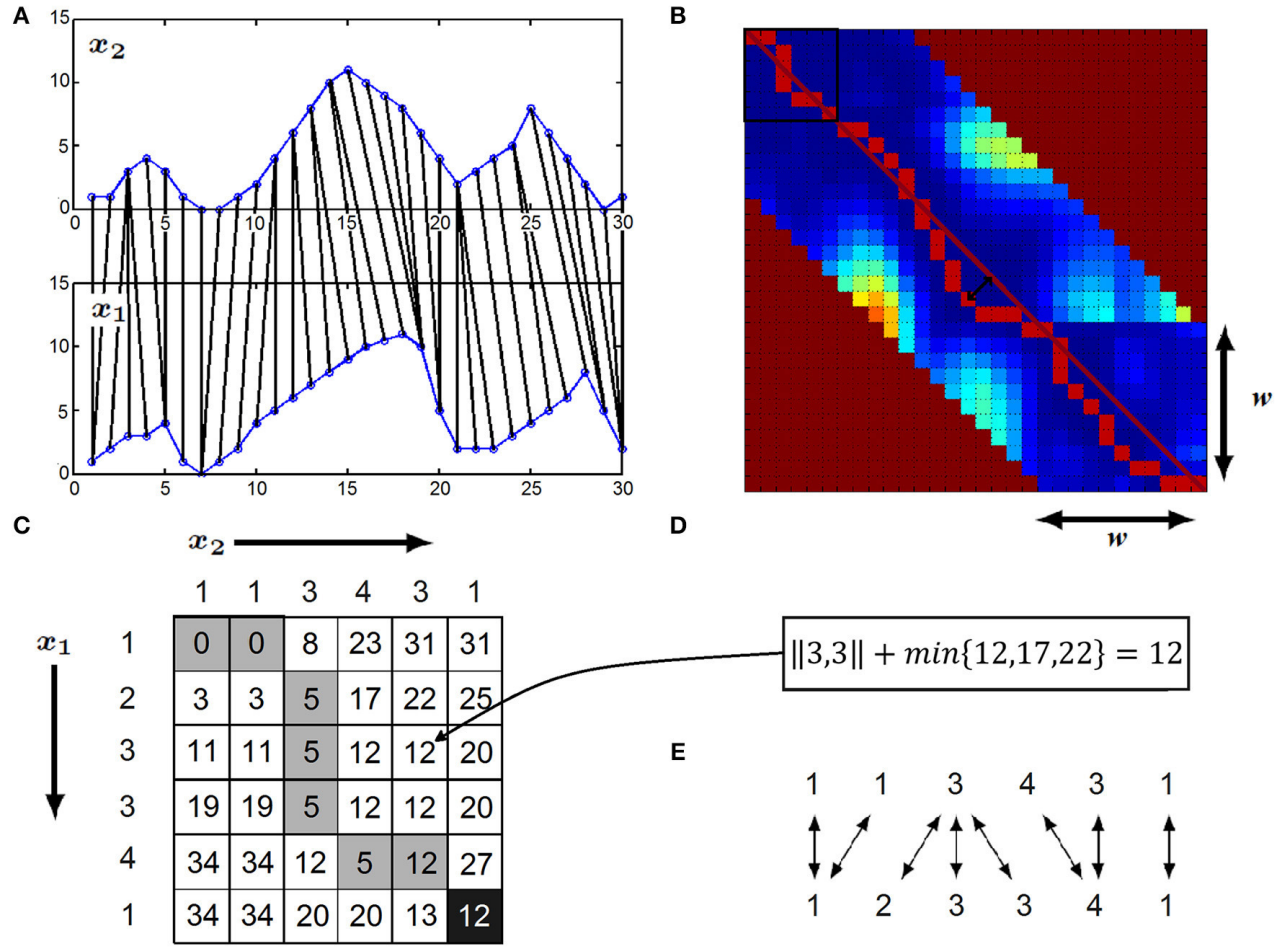
**(A)** *x*_1_ and *x*_2_ time series compared with DTW: the i-th element of *x*_1_ is elastically matched elastically matched to the appropriate element of *x*_2_. **(B)** The filled-out DTW matrix plotted as a heat-map (warmer color represents larger values), w denotes the size of the warping window, the maximal allowed time-lag between two matched time series element. The main diagonal is represented by the dark red line, while the warping path is plotted with red. The time-delay between the *x*_1_ and *x*_2_ time series at a given time-point is given by the warping path's deviation from the main diagonal (represented by the black arrows) **(C)** Calculation of DTW distance by filling out the DTW matrix: the example shows the first six element of *x*_1_ and first six element of *x*_1_ and *x*_2_ time series highlighted with the black rectangle in **(B)**. Elements of *x*_1_ corresponds to rows, while elements of *x*_2_ corresponds to columns of the matrix. The optimal warping path is highlighted with dark gray. **(D)** Formula to calculate entry (*i*,*j*)—in this example entry (3,5): squared distance of *x*_1_(*i*) and *x*_2_(*j*) plus the minimum of the matrix entries (*i*−1,*j*), (*i*−1,*j*−1), (*i*,j−1) **(E)** Optimal matching of the first six elements of *x*_1_ and *x*_2_ revealed by the DTW matrix.

Dynamic Time Warping algorithm uses elastic matching, which can correct for phase-shifts as well as distortions of the shape of the signal, as illustrated in Figure 1A. For example, the peak in *x*_1_ is matched to the peak in *x*_2_ when comparing the *x*_1_ and *x*_2_ with DTW (Tomašev et al., 2015). At the conceptual level, the calculation of the DTW distance of two time series *x*_1_ and *x*_2_ can be considered as transforming one of the time series into the other one, i.e., DTW is an edit distance. While doing that, each edit step is associated with a cost and the final DTW distance is the sum of the costs of the editing steps that are able to transform *x*_1_ into *x*_2_ with minimal total costs. In particular, two editing steps are possible: (i) the replacement of an element of *x*_1_ to an element of *x*_2_ and (ii) elongation of an element of *x*_1_ or *x*_2_. In both cases, some elements of the two time series are matched. The cost of a single editing operation is the difference between the matched elements of the two time series.

At the technical level, DTW distance is calculated by filling an *l*_1_-by-*l*_2_ matrix, called DTW matrix, where *l*_1_ and *l*_2_ refer to the length of time series *x*_1_ and *x*_2_, respectively. We use *DTW*(*i,j*) to denote the (*i*,*j*)-th entry of the aforementioned matrix. The (*i*,*j*)-th entry of the DTW matrix corresponds to the distance between the prefix of length *i* of *x*_1_ and the prefix of length *j* of *x*_2_ and it can be calculated according to Equation (1).

(1) $$DTW\left(i,j\right)=\{\begin{array}{c} \|x_{1}\left(i\right),x_{2}\left(j\right)\|+min\left\{DTW\left(i,j-1\right),DTW\left(i-1,j\right),DTW\;\left(i-1,j-1\right)\right\}if\;i,j>1\\ \|x_{1}\left(i\right),x_{2}\left(j\right)\|+DTW\left(i,j-1\right)\;if\;i=1,\;j>1\\ \begin{array}{c} \|x_{1}\left(i\right),x_{2}\left(j\right)\|+DTW\left(i-1,j\right)\;if\;j=1,\;i>1\\ \|x_{1}\left(i\right),x_{2}\left(j\right)\|\;if\;i=1,j=1 \end{array} \end{array}$$

As the (*i*,*j*)-th entry of the DTW matrix only depends on the (*i*−*1,j*−*1*)-th, (*i*−*1,j*)-th and (*i,j*−*1*)-th entries, the entries of the matrix can be calculated column-wise, beginning with the first entry of the first column, followed by the entries of the same column and the entries of subsequent columns, see Buza (2011) for details.

In order to ensure robustness against noise, we calculate the difference between two time series values *x*_1_(*i*) and *x*_2_(*j*) as $x_{1}\left(i\right),x_{2}\left(j\right)={\left(x_{1}\left(i\right)-x_{2}\left(j\right)\right)}^{2}$ and the distance of the two time series *x*_1_ and *x*_2_ is the square root of the (*l*_1_,*l*_2_)-th entry of the DTW matrix (see Figures 1C,D). In case if we assume that time-shifts between *x*_1_ and *x*_2_ have a limited size of *w* positions at most, i.e., the *i*-th element of time series *x*_1_ shall be matched to one of the elements of time series *x*_2_ between its (*i*−*w*)-th and (*i*+*w*)-th position, we calculate only the entries at most *w* positions far from the diagonal (see Figure 1B), *w* is called the warping window size.

After the DTW distance calculation, the optimal warping path can also be obtained from the DTW matrix (see Figures 1B,C) as follows: beginning with the (*l*_1_,*l*_2_)-th entry, we examine which entry of the DTW matrix lead to the minimum in Equation (1) and traverse the entries of the DTW matrix according to this minimum as long as we arrive at the (1,1)-st entry. This optimal warping path gives the matching between the positions of the two time series (Figure 1E).

### Application of dynamic time warping to fMRI data

The results of DTW analysis are strongly influenced by the choice of the warping window size, i.e., the maximal allowed time lag between the time series. The proper warping window size should be longer, than the longest expected time lag. As the high-pass filter used in resting-state studies has a cut-off frequency around 0.01 Hz, which corresponds to 100 s period and anticorrelations arise from time-delays of a half-period (50 s at 0.01 Hz), the 100 s warping window size is sufficient to detect anticorrelations of the lowest frequencies even with their potential time-delay fluctuations. On the individual subject's data, we examined how the choice of the warping window size effects the results. If we wish only to detect (supposedly) highly positively correlated areas, a window size of 20 s would be long enough, these experiments result in clearly visible homotopic and within-network connections. However, connections traditionally classified as anticorrelations (e.g., default mode and task-positive relations) usually exhibited time delay of 20–60 s, thus the proposed 100 s warping window is indeed long enough to capture these relationships as well.

**Figure 2:**
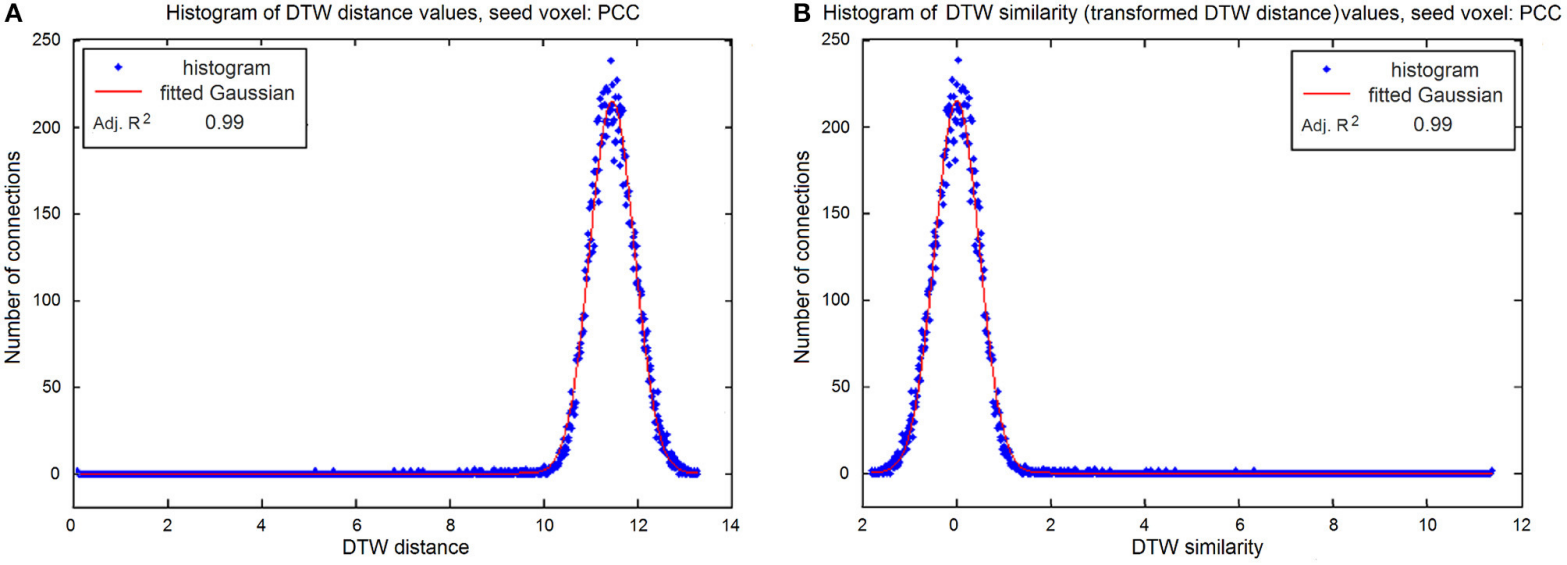
**(A)** Histogram of original DTW distances of the seed voxel (PCC) and the GM voxels, where zero distance means strong connection and high values indicate no relationship between the voxels, the histogram follows normal distribution with and adjusted *R*^2^ of 0.99. **(B)** Histogram of DTW similarity (transformed DTW) values: DTW distances are multiplied by −1 and then demeaned. The resulted histogram follows normal distribution with zero mean and adjusted *R*^2^ of 0.99.

There are important differences if we measure the strength of the relationship of two time series with DTW distance or linear correlation coefficients. Correlation coefficients ranges from −1 to +1, and the strength of the connection is determined by the coefficient's absolute value ranging from 0 (no correlation) to 1 (maximum correlation). DTW distance, on the other hand, is inherently positive and strong connection results in a distance close to 0 (no distance). To make the DTW distance measures comparable to that of the correlation coefficients, as well as to be able to perform parametric tests, we transformed our calculated DTW distances to similarity values. In practicality, the maximal DTW distance of two time-series is constrained by the range of the values in the time series, and the number of time points, i.e., when comparing time-series with zero mean and standard deviation of one, one can expect larger DTW distances, if the compared time-series are longer. In our case, for example, DTW values ranged between 0 and 14 (see Figure 2A), with a normally distributed peak around 11.5, which means that the distance between two unconnected voxel's time-series varies around 11.5. To transform distance to similarity (so the maximal value corresponds to the maximal connection strength), the DTW values were multiplied by −1. Then DTW values were demeaned, so that the potentially irrelevant DTW values are close to zero (see Figure 2B). From this follows that if there is no connection, the transformed DTW values will vary around zero, and follow a normal distribution, meaning the traditional one-sample *t*-test can be used to find statistically significant voxels. From now on, we refer to the transformed DTW values as DTW similarities throughout the paper.

DTW similarity measures the strength of functional connectivity similarly to the absolute value of the linear correlation coefficient, namely the higher the DTW similarity value the stronger the connection is. If two regions have a high positive DTW similarity value, their correlation can be either strong positive or strong negative, while negative DTW similarities mean that the two time series are as unrelated as possible. Importantly, the histogram of DTW similarities (Figure 2B) is characterized by a very long positive tail, which means that DTW is able to effectively differentiate between a diverse set of strong connections. This property makes DTW analysis especially suitable for connectivity pattern classification. On the other hand, it is important to note that DTW is not appropriate to determine whether the connection is “positive” or “negative.” However, this information can easily be derived by calculation of the correlation coefficient.

**Figure 3:**
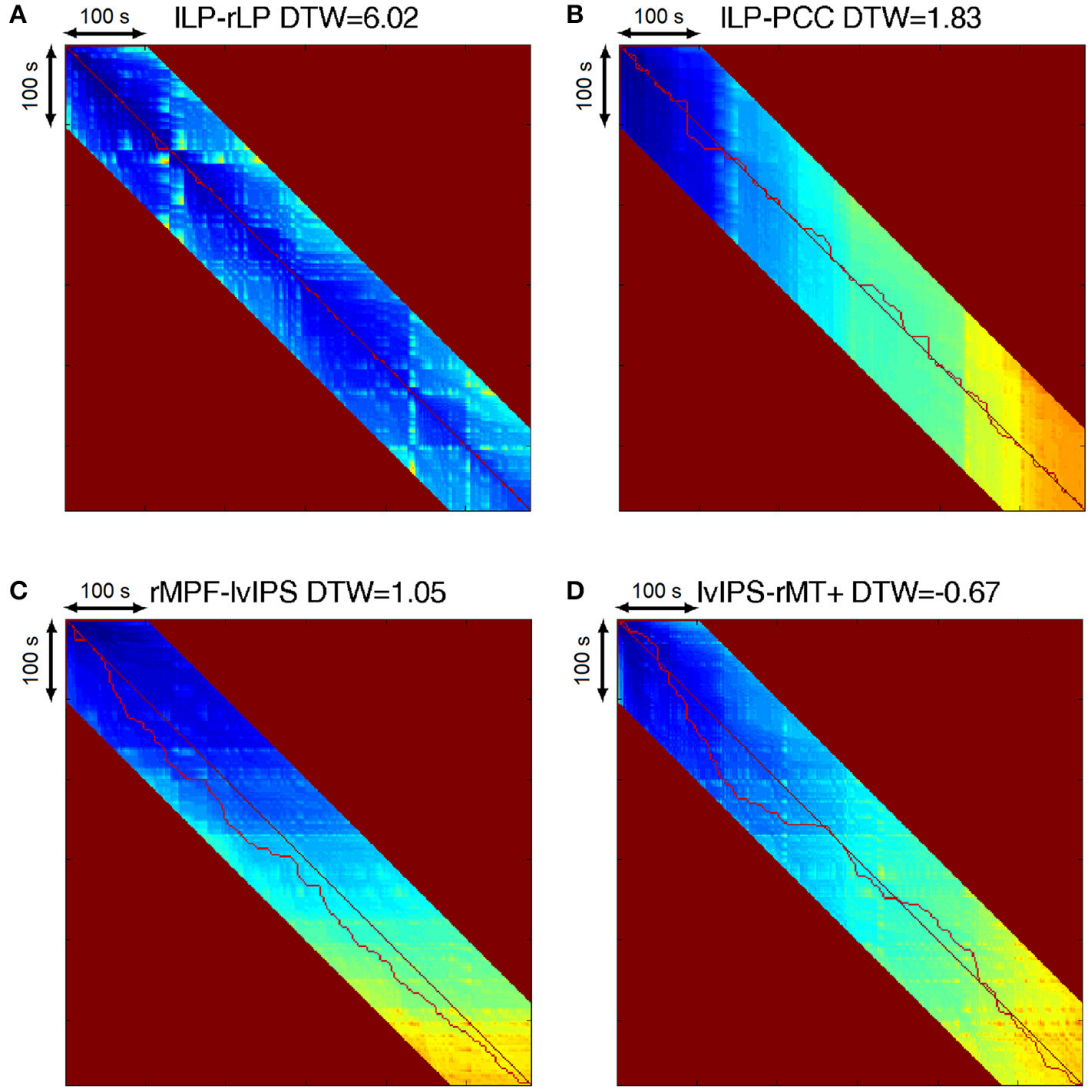
**(A)** Warping path of homotopic seeds left and right LP. The path follows almost exactly the diagonal of the DTW matrix, and the DTW similarity value is very high, indicating a strong connection. **(B)** Warping path of strongly correlating seeds left LP and PCC. The path follows the diagonal of the matrix, but timing jitters of 5–15 s appear in both directions, while the DTW similarity value still indicates a strong connection. **(C)** Warping path of anticorrelating seeds right MPF and left vIPS. The path follows a subdiagonal line representing a 30 s time-delay, with 5–15 s jitters, with a DTW similarity still significantly high. **(D)** Warping path of seeds with no significant connection, left vIPS and right MT+. The path has large roundabouts around the diagonal of the matrix, with amplitudes reaching 20–80 s in both directions. The DTW similarity is negative, indicating no relationship between the two voxels.

Figure 3 illustrates how DTW similarity describes connection strength, by showing some basic type of the connections, and the warping paths they generate. Between homotopic, and strongly correlating voxels, the warping path follows the diagonal of the DTW matrix, and DTW similarities are very high (Figures 3A,B), while relationships described as anticorrelation typically manifest as a relatively constant delay between the two time series (see Figure 3C), with also relatively high DTW similarity. The warping path between two unrelated signals exhibits a largely varying time-shifts in both positive and negative directions, and the DTW similarity value is around zero (see Figure 3D).

### Experiment 1–simulated data

#### Experiment 1. A–effects of transient interactions

Experiment 1.A demonstrates the behavior of DTW distance and correlation coefficient values when the interaction of time courses is transient. The simulated signals are 10 min long (600 s) with 2 s sampling rate and the time courses were filtered from white Gaussian noise with a band-pass filter using a combination of temporal high-pass (based on the regression of ninth-order discrete cosine transform basis set) and low-pass (bidirectional 12th-order Butterworth IIR) filters to retain signals only within the range of 0.009 and 0.08 Hz, the same frequency filter that is traditionally used for preprocessing resting state fMRI data. The empirical distribution of the DTW distance and correlation coefficient values of independent signals were constructed from 1,000 independently simulated signals' pairwise DTW distance and correlation coefficient value. In case of correlation coefficients we choose the 95 and 5 percentiles of the empirical distribution as significance threshold, while for DTW distances the threshold is the 5 percentile, as strong connection is reflected by DTW distance close to zero. We modeled transient interaction of time courses by copying a piece of the original signal into another independently generated noise signal, either in synchrony or with a given time delay. In the simulation we examined the significance of the calculated correlation coefficient and DTW values as a function of the length of the interaction and time delay.

#### Experiment 1.B–effect of the common noise on anticorrelations

Experiment 1.B demonstrates the robustness of DTW distance in the presence of superimposed common noise (representing the “global signal” component). We generated two perfectly anticorrelated sinusoid signal with 0.08 Hz frequency with independent frequency filtered white noise added, so the level of correlation is reduced from −1 to −0.86. We examined the changes in both correlation coefficient and DTW distance values as the function of the weight of linearly added common noise in the range of 0–50%. The noise signals, and the empirical null distribution were calculated from frequency filtered independent white Gaussian noise signals with the same method as in the previous simulation.

### Experiment 2–seed based connectivity analysis of rs-fMRI data

#### Subject

A right-handed female subject (aged 28 years) participated in the experiment and gave informed written consent in accordance with the protocols approved by Health Registration and Training Center (ENKK 006641/2016/OTIG), Budapest, Hungary. She did not have any history of neurological or psychiatric diseases, and had normal visual acuity.

Ten fMRI sessions were spread over 5 days (one morning and one afternoon). In each session two runs of 10-min-long closed-eyes resting-state runs were conducted.

#### Data acquisition

Data were collected at the Brain Imaging Centre of the Research Centre for Natural Sciences of the Hungarian Academy of Sciences (Budapest, Hungary) on a 3 T Siemens MAGNETOM Prisma scanner (Erlangen, Germany). High-resolution anatomical images were acquired using a T_1_-weighted 3D-MPRAGE sequence (1 mm isotropic voxels; TR = 2300 ms; TE = 3.03 ms; FOV = 256 mm; acceleration factor = 2). A total of 340 functional images over 10 min and 1.8 s were collected with a BOLD-sensitive ${\text{T}}_{2^{*}}$ weighted GRE-EPI sequence (3 mm isotropic voxels; TR = 1770 ms; TE = 30 ms; FOV = 204 mm; acceleration factor = 2). Thirty-three axial slices were acquired in an ascending acquisition order with a slice gap of 0.75 mm covering the whole brain.

#### Preprocessing

Preprocessing and analysis of the imaging data were performed using the SPM12 toolbox (Wellcome Trust Centre for Neuroimaging) as well as custom-made scripts running on MATLAB 2015a (The MathWorks Inc., Natick, MA, USA). In the single subject study all functional images were motion-corrected: EPI images from all sessions were spatially realigned to the mean image of the fourth session in order to register functional images from all sessions into a common space. Then, EPI images were spatially smoothed using an 8 mm full-width half maximum Gaussian filter. The anatomical T_1_ image was coregistered to the mean functional ${\text{T}}_{2^{*}}$ image also used during the realignment step. The coregistered T_1_ images were segmented using the unified segmentation and normalization tool of SPM12. The resulting gray matter (GM) mask was used to restrict statistical analysis of the EPI images to GM voxels; while white matter (WM) and cerebrospinal fluid (CSF) masks were used to extract nuisance signals that are unlikely to reflect neural activity in resting-state data. After regressing out the head-motion parameters, mean WM and mean CSF signals and their first five components obtained from PCA (Chai et al., 2012), residual time courses from all GM voxels were band-pass filtered as described in Section Experiment 1—Simulated Data.

As our aim was to study effects of GSReg, we preprocessed each dataset twice. In one case, in addition to the abovementioned preprocessing steps, we regressed mean time series of brain voxels out from the GM voxel time courses before band-pass filtering.

#### Seed-based connectivity analysis

Seed-based connectivity analysis was used to compare the within subject variance of three different metrics: DTW distance, conventional correlation, and cross-correlation analysis. Thirteen seed voxels were selected according to previous relevant studies (Fox et al., 2005; Chai et al., 2012). Five seeds are located in the DMN (PCC, left and right MPF, and LP), and eight seeds positioned in task-positive networks (left and right FEF, insula/FO, vIPS, MT+), for further details, see Supplementary Table 1.

In the single-subject data whole-brain correlation, maximal lagged cross-correlation, and DTW maps were generated based on four preprocessed resting-state datasets in each session: first run with GSReg, first run without GSReg, second run with GSReg, second run without GSReg. For each GM voxel, we estimated both the linear correlation and cross-correlation coefficients, and the DTW distance between the time course of the 13 seed voxels and the time course of the given voxel.

We calculated cross-correlation coefficients between the time course of the 13 seed voxels and the time course of the given voxel, with longest possible lag equal to the warping window size set for the DTW algorithm (see Section Application of DTW to fMRI Data). From the calculated cross-correlation values the maximal lagged correlation coefficient was selected as a descriptor of connectivity, similarly to the functional network connectivity (FCN) method described by Jafri et al. (2008), except we compared BOLD signals voxel-wise, and not component time-series assessed by ICA.

Before DTW distance calculation the compared time series were normalized, so that the mean of the normalized time series equals to zero and the standard deviation is one. This is in accordance with correlation and cross-correlation calculations, as these metrics perform normalization by definition.

Statistical parametric maps were derived via performing a voxel-wise one-sample *t*-test of the resulted connectivity maps using fixed-effect analysis.

### Experiment 3–whole-brain connectivity pattern classification

#### Subjects

Publicly available data from Consortium for Reliability and Reproducibility (CoRR; Zuo et al., 2014) LMU 1 dataset (Blautzik et al., 2013a,b) were used: 26 subjects [14 males, all right handed, age (mean ± *SD*): 24.2 ± 1.85 years]. Each subject participated in one fMRI session, containing at least five 455-s-long resting-state runs.

#### Data acquisition

The LMU1 dataset was collected at the Institute of Clinical Radiology, Ludwig-Maximilians-University, Munich, Germany, on a 3 T Philips Achieva scanner (Best, The Netherlands). High-resolution anatomical images were acquired for each subject using a T_1_-weighted 3D TFE sequence (1 mm isotropic voxels; TR = 2375 ms; FOV = 240 mm; acceleration factor = 2/2.5). A total of 180 functional images over 455 s were collected with a BOLD-sensitive T2^*^ weighted GRE-EPI sequence (3 mm slice thickness with 2.95 × 2.95 mm in-plane resolution; TR = 2500 ms; TE = 30 ms; FOV = 240 mm; acceleration factor = 3). Fifty-two axial slices were acquired in ascending acquisition order covering the whole brain. Further details are available on the website of the dataset (http://fcon_1000.projects.nitrc.org/indi/CoRR/html/lmu_1.html).

#### Preprocessing

In the group study, preprocessing, and data analysis were performed with the pipeline described in Section Preprocessing of Experiment 2 except that the realigned and coregistered images were transformed to the MNI-152 space using the transformation matrices generated during the segmentation and normalization of the anatomical images.

#### Classification method

To quantify the discrimination ability of functional connectivity metrics, we performed a whole-brain connectivity pattern based classification on the group study data. To calculate ROI based whole-brain functional connectivity we used a functional atlas of FIND Lab, consisting of 90 functional regions of interest (Shirer et al., 2012) to obtain 90 functionally meaningful averaged BOLD signals in each run. From this 90 time series we can calculate full connectivity matrices with correlation, maximal lag cross-correlation or with DTW distance leading to 90 × 89/2 = 4005 pairwise connectivity features for each of the aforementioned three connectivity metrics. We obtained these connectivity features for each of the 26 subjects and each of the five runs preprocessed with and without GSReg, leading to two classification datasets with 26 × 5 = 130 instances for each preprocessing pipeline with all the three connectivity metric. From the publicly available phenotypic data, gender was selected as classification target.

Classification was based on the LASSO regression (Tibshirani, 1996) method: a regularized regression approach that achieves inherent feature selection, which enables it to perform well even in case of high dimensional datasets, like fMRI data (Li et al., 2009; Ryali et al., 2010; Ng et al., 2012; Suk et al., 2013; Rosa et al., 2015). The LASSO's objective (Equation 2) is to find the parameter vector $\overset{⇀}{\theta }$ that minimizes the sum of squared errors and the regularization term:‘

(2) $$\vec{\theta }=arg\;{\text{min}}_{\vec{\theta }}\frac{1}{N}\|\vec{y}\;-X\vec{\theta }{\|}_{2}^{2}+\lambda \|\vec{\theta }{\|}_{1}$$

where *N* is the number of examples, *X* ∈ ℝ^*Nxd*^ matrix contains the instances, *d* is the number of features, $\overset{⇀}{y}\;\in \;{\mathbb{R}}^{N}$ contains the desired label values, $\overset{⇀}{\theta }\;\in \;{\mathbb{R}}^{d}$ is the parameter vector, and λ ∈ ℝ is a hyper parameter controlling the regularization, or equivalently, the sparsity of the resulting model.

We performed experiments according to a leave-subject-out cross-validation protocol, in each round of the cross-validation, the value of the hyper parameter λ, was determined using the training data only. In particular, we performed an internal leave-one-subject-out cross-validation on the training data in order to select the value of hyper parameter from the interval between 0.0005 and 0.5 that maximizes the accuracy of the current internal leave-subject-out cycle.

To obtain a threshold of significant classification, we generated a set of 100,000 random labeling with “coin-flipping” (50–50% of zero or one label), and calculated the accuracy values of these random classifications. The threshold of significance, i.e., the 95 percentile of accuracy values of this random classifier is 55.4%.

For a stricter threshold, we can train and test our classifier with the original datasets, but with permuted labels (permutation testing; Nichols and Holmes, 2002). The 95 percentile of accuracies were calculated from 1,000 random permutation of the labels separately for each dataset. The results are summarized in Table 1.

**Table 1:** **Ninety-five percentile of accuracies of the LASSO classifiers on randomly permuted labels based on correlation, cross-correlation, and DTW connectivity features, with both preprocessing pipelines (with and without GSReg)**.

	**95 percentile**	
	**With GSReg (%)**	**Without GSReg (%)**
Correlation based	64.2	63.1
Cross-correlation based	61.9	61.9
DTW based	60.1	60.7

## Results

### Experiment 1–simulated data

#### Experiment 1.A–effects of transient interactions

**Figure 4:**
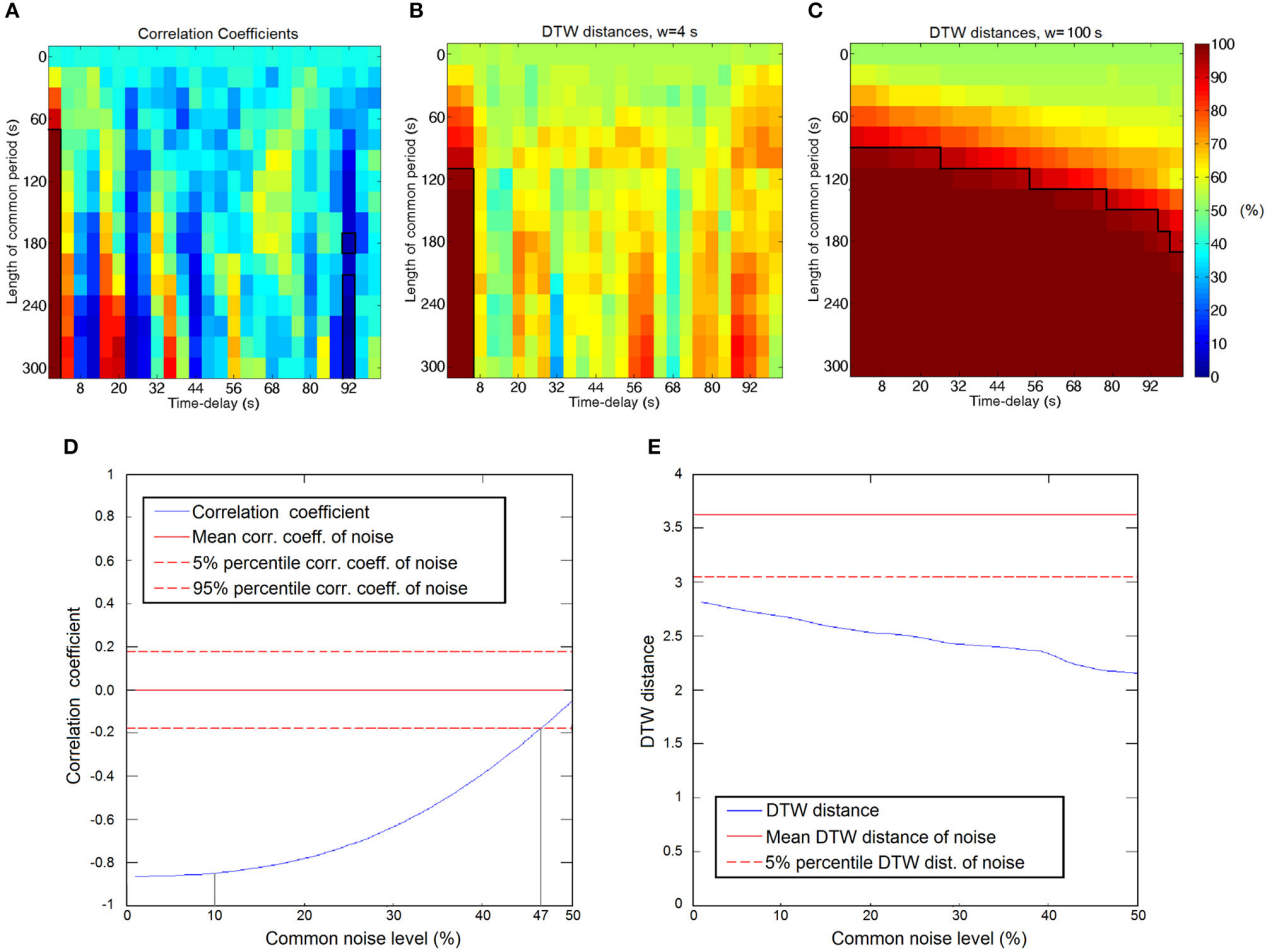
**(A–C)** Significance of connections between time courses as a function of time-delay and the length of the common period. Significant connections are framed, the thresholds are at the 5 and 95 percentiles of the empirical null-distribution. **(A)** Significance calculated from correlation coefficients. **(B)** Significance calculated from DTW distance with warping window size of 4 s. **(C)** Significance calculated from DTW distance with warping window size of 100 s. **(D,E)** Measurements' robustness for superimposed common noise. **(D)** Correlation coefficient as a function of the common noise level. Strongly anticorrelated signals become uncorrelated in the presence of the common noise. Hypothetical noise levels of 47 and 10% are denoted by the gray lines. **(E)** DTW distance values as a function of the common noise level. The connection is detected as significant at the 5 percentile threshold at the entire range of noise levels.

We applied simulation to demonstrate the feasibility of DTW distance to handle time-delays and dynamically changing interactions. We calculated DTW distance and correlation coefficient values as the function of the length of the interaction (the common signal) and time delay. As expected, correlation can efficiently capture even short term relationships between time courses, but only if the interaction is perfectly synchronized. As Figure 4A shows, significant positive correlations only arise at zero time-delay with coefficients ranging from 0.18 to 0.5, depending on the length of the shared signal, while some significant negative correlations appear at the 92 s time delay (0.011 Hz), if the common period is sufficiently long, however these negative correlations are weaker, with −0.12 as highest value. Figure 4B presents the significant connections found with DTW distance, with a very short, 4 s warping window size. The significant connections appear similarly to correlation, only at short (0–4 s) time-delays, while the choice of a larger window size (100 s) results in the diverse set of connections seen on Figure 4C. With sufficiently long warping window size, the DTW distance is able to detect transient interactions between time courses at any time delays, provided that the common period of the signals is long enough.

With this simulation we demonstrated that with appropriate parameters DTW distance is able to capture a wide range of dynamically changing relationships, which are undetectable, or have very low significance if examined with linear correlation.

#### Experiment 1.B—effect of the common noise on anticorrelations

Experiment 1.B demonstrates the robustness of DTW distance when common noise is present. If we consider the case of a measurement taken in the presence of relatively high level of common noise, e.g., global signal from respiration and cardiac movements, it is often the case (Desjardins et al., 2001), that we measure relatively weak negative correlations below the significance threshold, while after the GSReg step, these connections become significant (see Figure 4D; e.g., at hypothetical noise level of 47% before GSReg and 10% after GSReg). The DTW distance values, on the contrary, are passing significance threshold regardless of the presence of the common noise (Figure 4E), even though DTW values are changing with the increase of the noise level.

### Experiment 2–seed based connectivity analysis of rs–fMRI data

#### Experiment 2.A–effects of global signal regression

It is well-known that global signal regression (Murphy et al., 2009) may induce spurious negative correlations. To address the question of the DTW distance's sensitivity to the usage of GSReg, we applied two preprocessing pipelines to the measured data, one including the step of GSReg, and one without it, meaning from the ten session and two resting state measurement in each, we obtained forty datasets, to calculate DTW, correlation and cross-correlation maps from each seed voxel.

From the 40 DTW, correlation and cross-correlation maps per seed voxel, we calculated four T-maps to obtain statistical significance. The two T-maps are calculated from the ten datasets from each session's first resting state measurement preprocessed with, and without GSReg, and two T-maps are representing statistical significance of voxels in each session's second resting state measurement, also processed with, and without the GSReg step.

**Figure 5:**
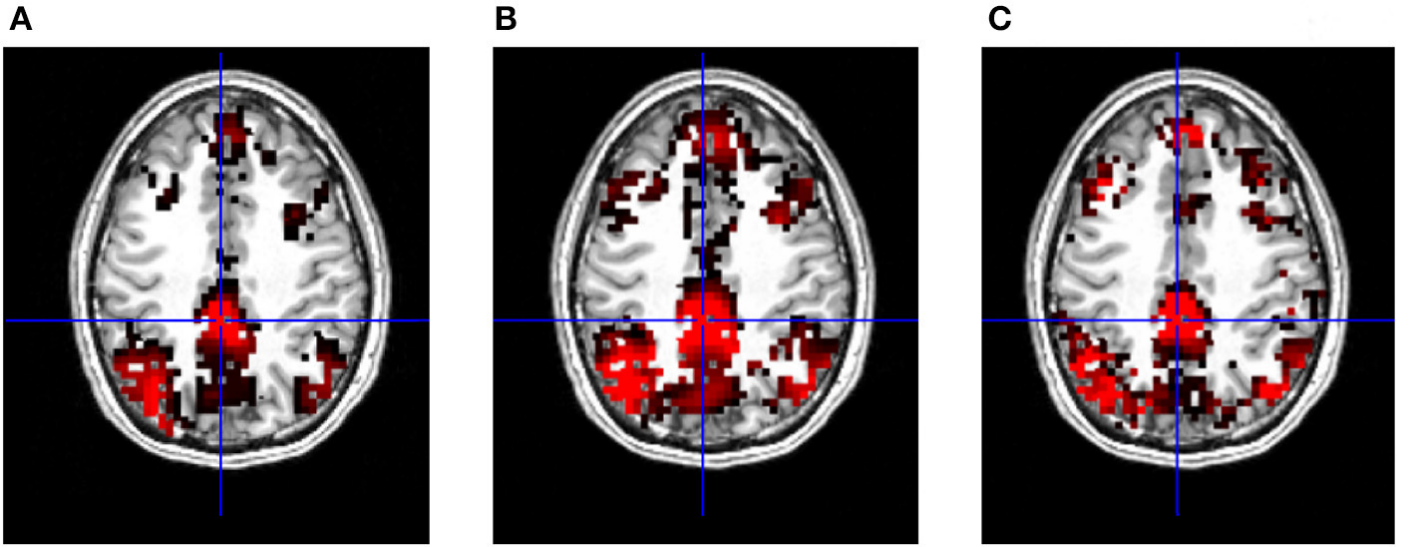
**T-maps of significant connectivity from the first measurements with GSReg, thresholded at FDR corrected *p* = 0.001**. **(A)** DTW based functional connectivity map with PCC seed, regions of the DMN are clearly visible. **(B)** Correlation based functional connectivity map with PCC seed, showing standard DMN regions. **(C)** Maximal lag cross-correlation based functional connectivity map with PCC seed, standard regions of the DMN are also visible.

Importantly, the results revealed that in terms of most significant voxels the T-maps look very similar in case of the three metrics (Figure 5). On the other hand, a closer inspection shows that maps produced with correlation tend to have larger, smoother clusters of activation, although the number of these clusters is usually lower, than on DTW based T-maps (Figure 5), maximal lag cross-correlation based maps are also smoother than DTW maps, however the cluster size is reduced compared to traditional correlation.

Next, we investigated the distance metric's sensitivity to the usage of global signal regression, by calculating the number of voxels that are passing threshold with only one preprocessing pipeline at a given significance level. We expected to find DTW distance's statistical results more coherent in the areas, where correlation (or cross-correlation) is known to produce unreliable significance in anticorrelations.

**Figure 6:**
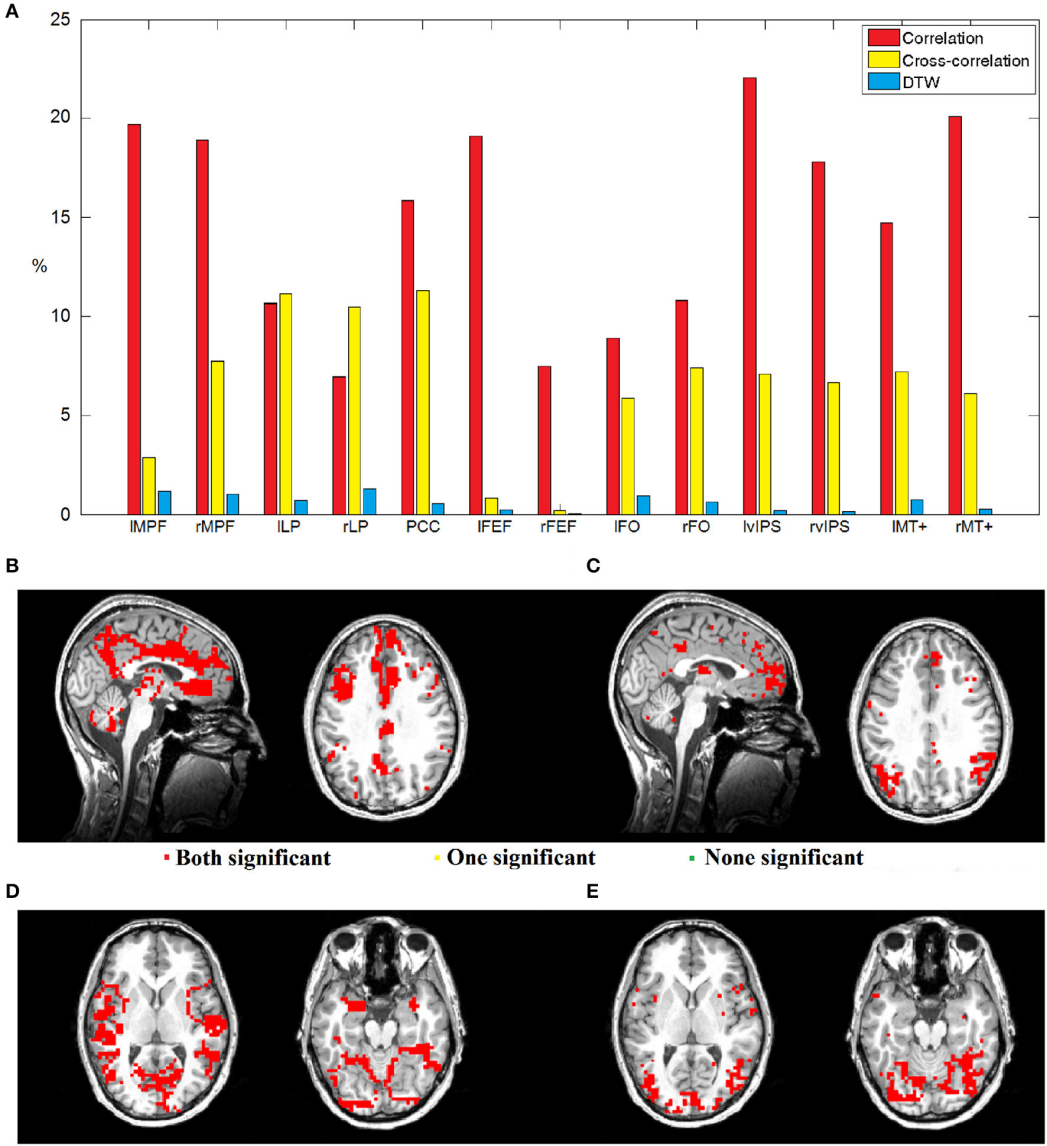
**(A)** Bar chart showing the percentages of voxels with instable significance considering the two preprocessing pipelines from altogether 19,375 GM voxels. Red bars represent the voxels of dubious negative correlations, yellow bars represent the voxels of dubious negative values of maximal lag cross-correlation, while blue bars denote instable voxels with DTW calculation. **(B–E)** Dubiously anticorrelating voxels mapped on the brain. **(B)** Areas of dubious zero-lag anticorrelations with seed voxel right vIPS, critical voxels are centered around nodes of the DMN. Voxels in red were found to be significantly connected with DTW, while green voxels showed no connection, and the interpretation of yellow ones is dubious even with DTW. As one can see, the areas of dubious anticorrelations were found to be connected with DTW measures. **(C)** Areas of dubious anticorrelations from maximal lag cross-correlation with seed voxel right vIPS, critical voxels are centered around nodes of the DMN. **(D)** Areas of dubious zero-lag anticorrelations with seed voxel PCC. **(E)** Areas of dubious anticorrelations from maximal lag cross-correlation with seed voxel PCC.

Given the thirteen seed voxels described above, we analyzed T-maps thresholded with FDR corrected *p* = 0.05, and in case of both correlation maps, we calculated the number of voxels that were significantly negatively correlated when preprocessing included GSReg, but were no more significant when we skipped this step. With DTW based T-maps we simply counted voxels, which passed the threshold with only one preprocessing pipeline (See Figure 6A). Since the number of critical voxels calculated with DTW is only around 10% of the number of dubious voxels calculated with correlation and 20% calculated with cross-correlation, DTW distance seems to be the most robust measure from the point of view of GSReg. Also, in areas where either correlation or cross-correlation based statistics results in possibly deceptive or unreliable anticorrelations, DTW behaves coherently.

Figures 6B–E shows the areas of dubious anticorrelations. In case of a seed taken from a task-related network, the dubious voxels are usually around the cores of the default mode network (Figures 6B,C), while DMN seeds tend to have dubious negative relationships with several task-induced networks (Figures 6D,E). As one can see, most of these questionable voxels show statistical significance with DTW similarity regardless of the preprocessing pipeline.

Our results clearly showed that DTW distance is the most robust measure with respect to global signal regression, even compared with maximal lag cross-correlation. Although the number of voxels with doubtful correlation statistics (negative correlation emerge only after GSReg) in cross-correlation based maps is usually much lower than the number of questionable voxels selected by zero-lag correlation, DTW produces 80% less spurious statistics than cross-correlation in almost all seeds.

#### Experiment 2.B–robustness in multiple measurements

**Figure 7:**
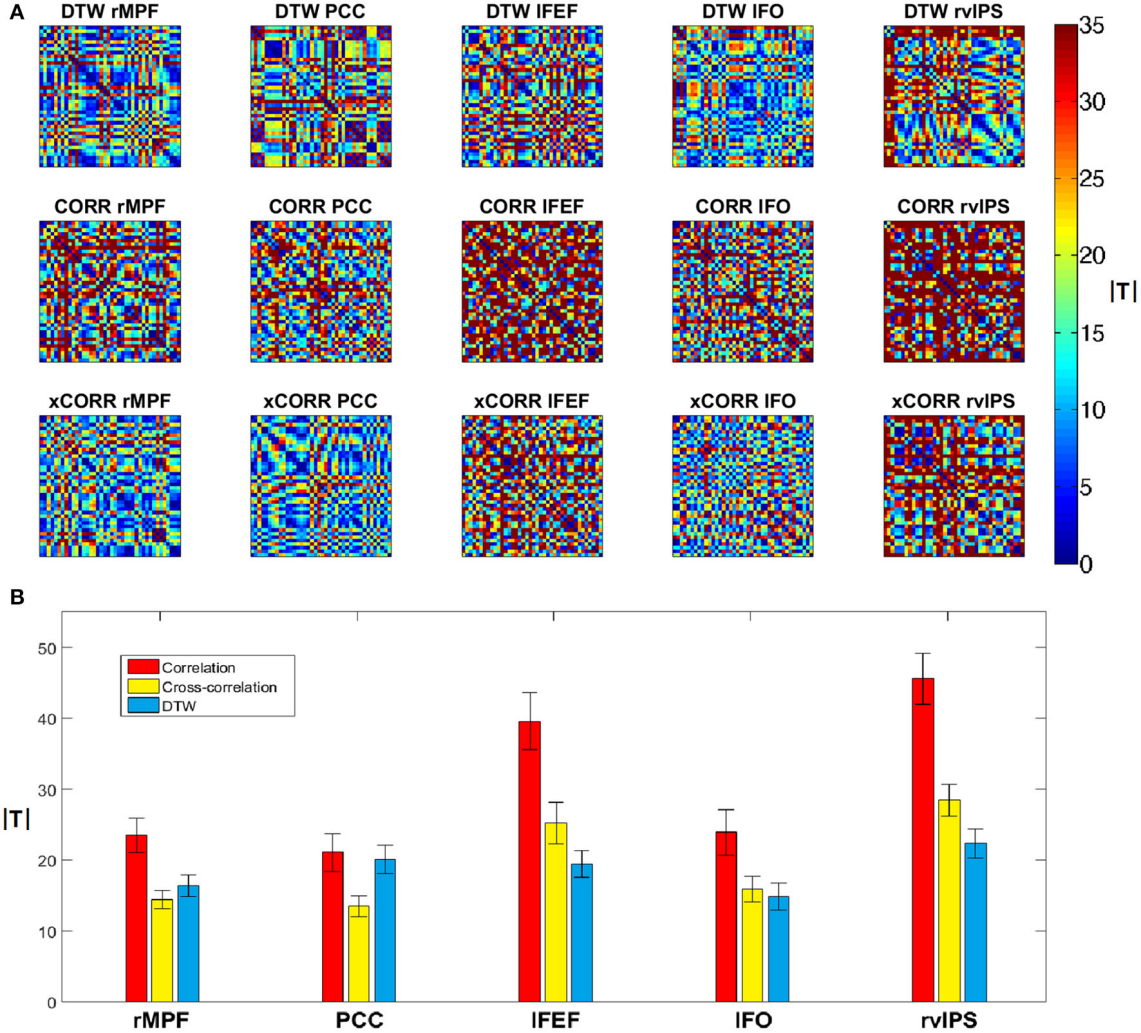
**Variability of DTW, correlation (CORR) and cross-correlation (xCORR) maps on the level of individual measurements**. For better visibility, the results are only plotted for five selected seed voxels (rMPF, PCC, lFEF, lFO, rvIPS). For the results of all thirteen seed voxels, see Supplementary Figure 1. **(A)** Absolute *T*-values from paired *T*-tests of connectivity maps thresholded at FDR corrected *p* = 0.05 for the five selected seed voxels. The order of comparison in each matrix is: first session's first run preprocessed with GSReg, first session's second run preprocessed with GSReg, first session's first run preprocessed without GSReg, first session's second run preprocessed without GSReg, followed by the second session's data and so on. **(B)** Average and standard deviation of absolute *T*-values plotted individually in the matrices of **(A)**. *T*-values of correlation are significantly higher than *T*-values of DTW in most seeds, while *T*-values of cross-correlation are usually comparable to *T*-values of DTW (even lower in case of DMN seeds).

Next we compared the robustness of the three different connectivity metrics across multiple measurements. We used a paired *T*-test to compare DTW similarity, correlation coefficient and maximal lag cross-correlation values between each dataset pair from the forty datasets (from the twenty repeated measurements, preprocessed both with and without GSReg), e.g., we compare two PCC seed based DTW similarity maps by calculating the *T*-value from the paired *T*-test on the two significance thresholded DTW similarity maps. The absolute *T*-values should be low, if the connectivity strength is the same between measurements, or different preprocessing pipelines. After calculating the absolute *T*-values from the paired *T*-tests, we can plot these differences on forty-by-forty matrices (see Figure 7A), since we had ten sessions, with two resting state runs each, and we obtained four different datasets from these, with the two separate preprocessing pipelines.

This experiment indicates that zero-lag correlation is the least robust from the three metrics, however the robustness of DTW and cross-correlation heavily depends on the choice of the seed voxel (see Figure 7B).

### Experiment 3–whole-brain connectivity pattern classification

To test the sensitivity of the DTW analysis to group differences in connectivity patters, we applied the same classifier to DTW, zero-lag correlation and maximal lag cross-correlation based feature sets. We trained the classifier to infer the participants' gender from the connectivity strengths calculated with either correlation, cross-correlation or DTW. The accuracy of the LASSO classifiers are summarized in Table 2.

**Table 2:** **Accuracy of the LASSO classifiers in gender identification based on correlation, cross-correlation, and DTW connectivity features, with both preprocessing pipelines (with and without GSReg)**.

	**With GSReg (%)**	**Without GSReg (%)**
Correlation based	60.0	44.6
Cross-correlation based	45.4	50.0
DTW based	68.5	66.9

From the results in Table 2 it is clear that DTW distance based classification produced the highest accuracies both with and without GSReg. Compared to the baseline classifier (random guessing) the LASSO classifier was able to achieve significant results in case of three feature sets: the two DTW distance based feature sets (with and without GSReg) as well as the correlation based feature set with GSReg. Interestingly, the sensitivity of maximal lag cross-correlation for group differences was found to be lower than that obtained for DTW and zero-lag correlation analysis, as neither cross correlation based feature sets resulted in significant classification accuracy.

The advantage of DTW distance based features is even more pronounced when we compare the resulting accuracies to the significance thresholds obtained from permutation testing (Table 1). In this case only the two DTW based feature sets lead to significant classification results, as neither zero lag nor maximal lag correlation features can outperform the 95 percentile accuracies. This results imply that DTW distance is a suitable descriptor of connectivity for classification in case of both preprocessing pipelines.

### Summary of the results

Taken together, our results revealed (see Table 3) that DTW measures of rs-fMRI functional connectivity are robust with respect to global signal regression and across multiple measurements. Furthermore, we also found superior sensitivity of the DTW analysis to group differences by showing that DTW based classifiers outperform the zero-lag correlation and maximal lag cross-correlation based classifiers.

**Table 3:** **Summary of the studied aspects of the three metrics and their performances**.

	**DTW**	**Correlation**	**Cross-correlation**
Robustness for GSReg	High	Low	Medium
Robustness in multiple measurements	Medium	Low	Medium
Sensitivity for group differences	High	Medium	Low

## Discussion

The results of the present study provide evidence that DTW is applicable as a distance metric of fMRI time series. We developed tools to calculate single seed connectivity maps and whole-brain connectivity matrix based on DTW distance. First, an appropriate warping window parameter (100 s) for DTW distance calculation was determined, and an apt transformation was developed for DTW distance values, that yields DTW similarity values interpretable with standard statistical analysis tools used in traditional correlation based studies. The obtained DTW value as a single scalar measure is suitable to describe functional connectivity strength similarly to traditional SCA or ICA methods. Next, we showed that seed based statistical maps produced based on DTW similarity are very similar to those obtained based on correlation or cross-correlation values. Due to the fact that, as our simulations demonstrated (Experiment 1.A), DTW distance and similarity is capable to capture more complex relationships than linear correlation, DTW based T-maps showed more clusters that are slightly better localized.

The strength of static functional connectivity between brain regions in case of an individual brain is expected to be stable across repeated measurements (Shirer et al., 2013). However, previous research showed that resting state correlations exhibit substantial temporal instability due to changing brain states (Chang and Glover, 2010; Allen et al., 2014; Chen et al., 2015), which implies that a metric that can capture and effectively handle non-stationery dynamics and autocorrelations would be required to achieve robust estimation of static functional connectivity. For this reason, we predicted that because DTW is able to handle even non-stationary time-lags and behaves more stable against superimposed common noise, the derived connectivity strength measurements will be more robust in case of DTW as compared to correlation analysis. The results of our experiments on simulated data (Experiment 1) and on data from real fMRI measurements (Experiment 2) provided support for our prediction.

We examined robustness from two aspects, Experiment 1.B and Experiment 2.A analyzed how the DTW algorithm handles the issue of global noise components. As we pointed out in the introduction, the presence of common noise (e.g., physiologic, represented by global signal) in the time series datasets strongly influences the functional connectivity calculated from correlation coefficients, while connectivity measured with DTW is less affected. Also, DTW can effectively capture intermittent irregular positive (in-phase) and negative (out-of-phase, in the range of 0–100 s delay depending on the parameterization) coherence between network nodes characterized by weak negative (usually bellow threshold) correlation coefficients as demonstrated in Experiment 1 with simulated data. Based on these properties, functional connectivity calculated from DTW similarity is shown to be more robust, and significantly less sensitive to global signal regression than standard SCA and maximal lag cross-correlation analysis. While—in accordance with the literature (Chang and Glover, 2009; Fox et al., 2009; Murphy et al., 2009)—GSReg substantially increase the extent of areas showing anticorrelating patterns between DMN and TP nodes, statistical maps based on DTW similarity calculation hardly change at all. More importantly, majority of the ROI pairs/voxels that are strongly influenced by the preprocessing strategy,-i.e., dubious significant negative correlation appears only after GSReg- can be identified with DTW regardless of the preprocessing.

In Experiment 2.B, we examined robustness in multiple measurements through the stability of seed-based networks resulting from DTW, correlation and cross-correlation calculation in twenty repeated resting-state runs measured within a week preprocessed with and without global signal regression. We were able to demonstrate that the variability of both DTW similarities and cross-correlation coefficients is substantially smaller than variability of zero-lag correlation coefficients, however we found that the difference between the stability of DTW and cross-correlation maps depends on the choice of the seed voxel.

Taken together, our experimental results revealed that DTW technique yields more robust results across distinct fMRI sessions and different preprocessing pipelines than the traditionally used SCA method, which is a desirable property primarily in longitudinal studies. The reliability of functional connectivity measures is a critically important question since there is a strong need for a robust biomarker to monitor mental diseases through a task-free measurement (Liu et al., 2008; Zuo et al., 2010; Zuo and Xing, 2014; Griffanti et al., 2016).

However, a biomarker's usefulness is characterized by not only its robustness, but its sensitivity for group differences. Therefore, in Experiment 3 we tested the DTW metric's discriminative ability in a whole-brain connectivity pattern based classification paradigm. The results revealed that robustness of the DTW metric does not compromise its sensitivity for group differences. We showed that DTW based classifiers considerably outperform the correlation and the maximal lag cross-correlation based classifiers. In contrast, the cross-correlation based paradigm seems to gain robustness at the expense of sensitivity for group differences. The higher accuracy produced by the DTW based classifiers implies that DTW indeed grasps important information about the group differences, presumably because DTW distance can describe a more diverse set of connections than correlation (Experiment 1.A) and DTW differentiates well between strong connections (Figure 2). The superiority of DTW based classifiers were demonstrated in ADHD and cannabis classification studies as well (Meszlényi et al., 2016a,b).

Another advantage of DTW that should be a subject of future research is that through the in-depth analysis of the warping paths we gain information of the dynamic changes in the relationship of the voxels. Neural signals have been shown to form dynamically changing adaptive patterns of activation over various time scales (Phillips et al., 2010; Principles of Brain Dynamics: Global State Interactions, 2012) Yet, even if the range of connectivity variation does not exceed that which can occur by chance, it may still be the case that the time-course of connectivity fluctuation tracks meaningful neural phenomena, as would be predicted from electrophysiological data (de Pasquale et al., 2010). Therefore, the shape of the warping path can hold valuable information of the relationship of the two time series (see Figure 3), just as DTW distance itself. This property of the DTW algorithm may serve as a basis for further applications on the field of neuroimaging, since beside the average connectivity strength characterized by DTW similarity, the warping path itself can reveal dynamic changes and directionality in the relationship of two time series. There is a lot of combination by which the different characteristics of a given warping path can be summarized by a single scalar value (e.g., number of diagonal crossings, maximal or average distance from the diagonal, etc.), for a classification experiment based on the length of the warping path see Meszlényi et al. (2016a).

Also an interesting area of use would be to apply DTW for task-based, and particularly event-related paradigms, since it is known, that the timing, and shape of the hemodynamic response function is different, not only between subjects, but between brain regions as well. Therefore, the DTW distance of the theoretical model's time series, and the measured signals can hold more accurate information, than the parameter set calculated from the general linear model.

Although the DTW approach has several advantageous features in functional connectivity calculations, it is important to note its limitations as well. The most prominent drawback of the algorithm is its computational complexity. While simple zero-lag correlation calculation's computation time increases linearly with the number of time-points, in case of DTW distance calculation, it is linearly dependent on the number of entries that are filled in the DTW matrix. Nevertheless, the calculation of whole-brain connectivity matrices or seed-based connectivity maps is easily parallelizable, thus the DTW calculation's computational cost can be reduced. When choosing the DTW approach for functional connectivity calculation, one should also consider that while correlation coefficients range between −1 and 1 by definition, the upper limit of DTW distance values depends on the length of the time-series we compare. Therefore, to interpret single DTW distance values, we need to know the underlying distribution as well (see Figure 2A) and compare the DTW distance in question to the DTW distances of independent time-series of the same length. This limitation can also affect comparison of different experiments. In principle, the correlation coefficients can be directly compared between two experiments with different time-series length, while DTW distances or similarities have to be normalized according to the length of the measurements before such comparison, e.g., by simply dividing the DTW distance values with the number of time-points, or by scaling the distribution of DTW similarities in both experiments so that the Gaussian peak has not only zero mean, but its variance equals one.

Since the dynamic properties of functional connectivity have drawn increasing attention, new metrics beside traditional SCA and ICA methods have been developed (Chang and Glover, 2010; Kiviniemi et al., 2011). Dynamic Time Warping distance and path based analysis fits in this trend by handling non-stationary time-lags on different scales, from 2 to 100 s. While sliding window approaches still search for zero-lag synchrony in shorter time periods, time-frequency coherence analysis strategies are able to identify time-lags as phase differences. The wavelet transform based method proposed by Chang and Glover (2010) indicates anticorrelations arise from 180° phase difference, which results in a time-lag above 30 s in the frequency range around 0.01 Hz, and that these anticorrelations are usually not stable during the whole scan. This is in accordance with our preliminary findings in warping path analysis, showing that very strong anticorrelating relationships have relatively constant time-lags around 30 s, however weaker negative relationships showed more fluctuations. An important property of DTW based calculation is, in contrast to e.g., the wavelet transform based approach, that the DTW similarity itself is a straightforwardly interpretable scalar measure that can be used in statistical analysis, while it still holds information of the dynamics of the examined relationship. Therefore, DTW could bridge the gap between traditional stationary methods and new dynamic approaches. DTW based functional connectivity calculation as stated above, results in a more stable description of the brain networks, than correlation based analysis. This means that the main fields of application of our method are longitudinal, or follow-up studies, and measurements, where individual results should hold valuable information, like biomarker research and classification experiments.

## Conclusion

This study shows that DTW is applicable as a distance metric of fMRI time series. Using simulated fMRI datasets we found that DTW is able to capture a wide range of interactions and it is less sensitive to linearly combined common noise in the data then zero-lag correlation analysis. The analysis of seed-based functional connectivity measurements using DTW revealed network structures well-known from conventional measurements, while DTW results in more stable connectivity strength on the level of individual distance values, even though its sensitivity for group differences is higher than correlation's as the classification study implies. Our findings show that analyzing resting state functional connectivity using DTW leads to higher performance in classification tasks, while its stability suggests DTW might be an efficient new approach to uncover changes in brain functional networks in longitudinal studies involving multiple repeated measurements.

## Ethics statement

The protocols were approved by Health Registration and Training Center (ENKK 006641/2016/OTIG), Budapest, Hungary, and the subject gave informed written consent in accordance with the Declaration of Helsinki.

## Author contributions

All coauthors contributed to this study: RM, PH, KB, VG, and ZV.

## Funding

This work was supported by a grant from the Hungarian Brain Research Program (KTIA_13_NAP–A–I/18) to ZV. KB was supported by the National Research, Development and Innovation Office—NKFIH PD 111710 and the János Bolyai Research Scholarship of the Hungarian Academy of Sciences.

### Conflict of interest statement

The authors declare that the research was conducted in the absence of any commercial or financial relationships that could be construed as a potential conflict of interest.
