# Supplementary material for: Resting State fMRI Functional Connectivity Analysis Using Dynamic Time Warping
Source: Front Neurosci. 2017 Feb 17;11:75. doi: 10.3389/fnins.2017.00075 (PMC5313507; doi:10.3389/fnins.2017.00075)
Supplement: Supplementary file 1 [file Table1.PDF]

Supplementary Material

Resting state fMRI functional connectivity analysis using Dynamic Time Warping

Regina Meszlényi*, Petra Hermann, Krisztian Buza, Viktor Gál and Zoltán Vidnyánszky

*** Correspondence:** Regina Meszlényi: meszlenyi.regina@ttk.mta.hu

MNI	lMPF	rMPF	lLP	rLP	PCC	lFEF	rFEF	rFO	lFO	lvIPS	rvIPS	lMT+	rMT+
x	-3	2	-47	52	-3	-24	27	-48	48	-26	34	-48	54
y	44	57	-70	-71	-36	-17	-11	8	6	-84	-85	-69	-62
z	-2	24	37	38	39	68	59	5	12	26	29	-10	-16

Supplementary Table 1. MNI coordinates of the thirteen seed voxels used in Experiment 2: left and right medial prefrontal cortices (MPF), left and right lateral parietal cortices (LP), posterior cingulate cortex (PCC), left and right frontal eye fields (FEF), left and right frontal operculum (FO), left and right ventral intraparietal sulci (vIPS) and left and right middle temporal complex (MT+)
