# Supplementary material for: Resting State fMRI Functional Connectivity Analysis Using Dynamic Time Warping
Source: Front Neurosci. 2017 Feb 17;11:75. doi: 10.3389/fnins.2017.00075 (PMC5313507; doi:10.3389/fnins.2017.00075)
Supplement: Supplementary file 2 [file Image1.PDF]

Supplementary Material

Resting state fMRI functional connectivity analysis using Dynamic Time Warping

Regina Meszlényi*, **Petra Hermann, Krisztian Buza, Viktor Gál and Zoltán Vidnyánszky**

*** Correspondence:** Regina Meszlényi: meszlenyi.regina@ttk.mta.hu

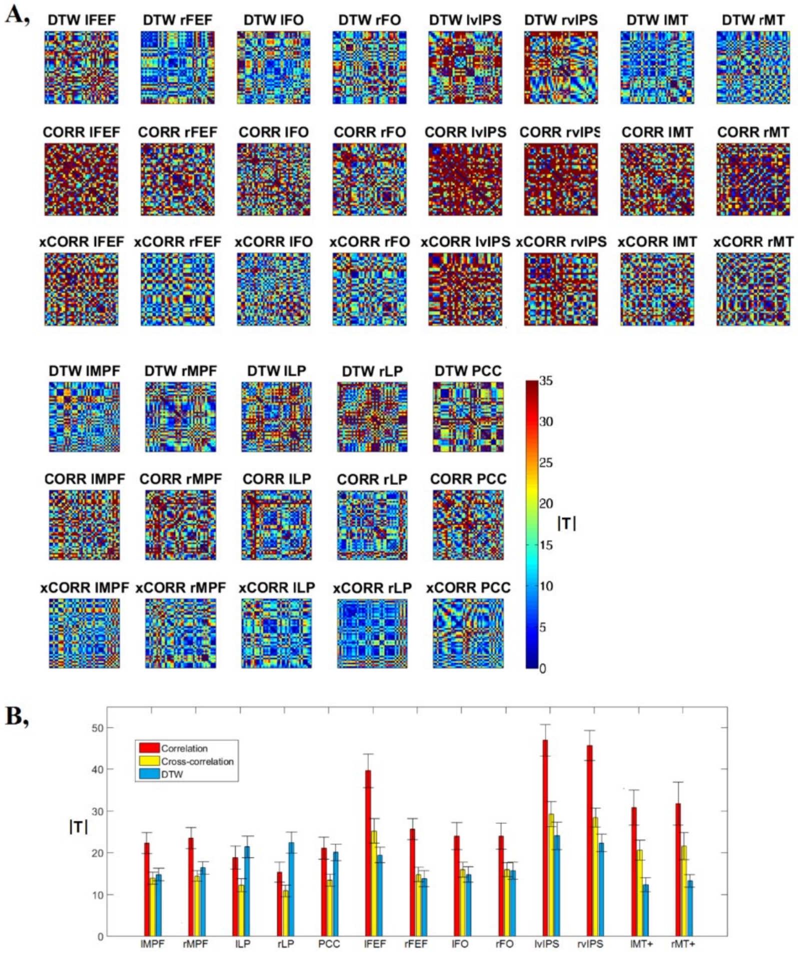

Supplementary Figure 1. Variability of DTW, correlation (CORR) and cross-correlation (xCORR) maps on the level of individual measurements, for all thirteen seed voxels. A, Absolute T values from paired T-tests of connectivity maps thresholded at FDR corrected p=0.05. The order of comparison in each matrix is: first session's first run preprocessed with GSReg, first session's second run preprocessed with GSReg, first session's first run preprocessed without GSReg, first session's second run preprocessed without GSReg, followed by the second session's data and so on. B, Average and standard deviation of absolute T values plotted individually in the matrices of Supplementary Fig. 1.A. T values of correlation are significantly higher than T values of DTW in most seeds, while T values of cross-correlation are usually comparable to T values of DTW (even lower in case of DMN seeds).
